# Charcot Neuroarthropathy of the Shoulder Caused by Cervical Spondylotic Myelopathy: A Case Report and Literature Review

**DOI:** 10.7759/cureus.64776

**Published:** 2024-07-17

**Authors:** Min Kyu Park, Neil Ashwood, Andrew P Dekker, Adam T Stammer, Gur Aziz Sidhu

**Affiliations:** 1 Trauma and Orthopaedics, University of Leicester, Leicester, GBR; 2 Trauma and Orthopaedics, University Hospitals Derby and Burton, Derby, GBR; 3 Trauma and Orthopaedic Surgery, University Hospitals Derby and Burton, Burton-on-Trent, GBR; 4 Orthopaedic Surgery, Burton Hospital, Burton, GBR; 5 Trauma and Orthopaedics, University Hospitals Derby and Burton, Burton on Trent, GBR

**Keywords:** charcot neuroarthopathy, syringomyelia, reverse shoulder arthoplasty, cervical spondylotic myelopathy (csm), charcot's joint

## Abstract

Charcot neuroarthropathy (CN) is a chronic degenerative disorder of bones and joints, mostly associated with diabetes mellitus and human immunodeficiency virus. CN of the upper limb is rare, with only 58 case reports identified on PubMed with the majority of cases being closely associated with syringomyelia. Very rarely, cervical spondylotic myelopathy (CSM) is associated with CN of the upper limb; with very few literature reporting this association. This case report presents a rare case of Charcot arthropathy of the shoulder caused by CSM.

A 57-year-old female presented to the emergency department following trauma to the right shoulder. On clinical examination, there was evidence of tenderness, extensive swelling, and bruising with a lack of range of motion along with numbness in the right arm and legs. Through radiographic and laboratory investigations, a diagnosis of CN secondary to CSM was made. A reverse total shoulder arthroplasty was performed however, this was complicated at two weeks with an atraumatic glenoid fracture and dislocation. First-stage revision surgery was then performed to allow fracture healing pending second-stage revision surgery.

This report provides insight into the very rare possibility of the association of CN of the shoulder with CSM. A review of the literature suggests reverse shoulder arthroplasty is the gold standard for cases of severe bone and soft tissue damage. When undergoing investigations for Charcot neuroarthropathy, physicians must undertake a full detailed history along with a detailed neurological examination and imaging of the cervical spine to not miss the association with CSM.

## Introduction

Charcot neuroarthropathy is a chronic degenerative disorder of the bone structures and joints that develops due to the disturbance of the innervation of joints [1]. It is associated with a variety of different conditions most notably diabetes mellitus, human immunodeficiency virus (HIV), syphilis, and syringomyelia, with the latter being the leading cause of neuroarthropathy in the upper limbs [2].

To this date, there have only been three published reports that report cases of Charcot arthropathy due to cervical spondylotic myelopathy, with only one presenting with neuropathy of the shoulder [3-5]. This article describes a case of Charcot neuropathic arthropathy of the shoulder secondary to cervical spondylotic myelopathy to provide insight into the rare cause of this degenerative disorder.

## Case presentation

A 57-year-old female with a past medical history of anxiety presented to the emergency department of Queens Hospital Burton with pain in the right shoulder following a fall. On examination, tenderness was felt at the proximal arm and shoulder along with extensive swelling and bruising. There was a restricted range of motion along with pain.

**Figure 1:**
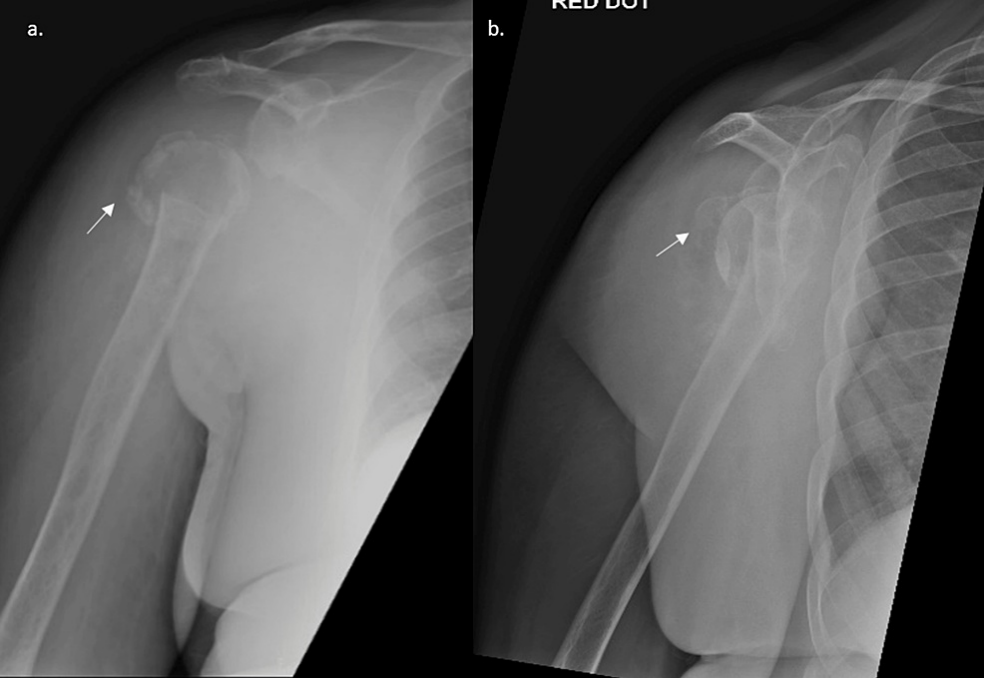
Pre-operative radiographs a. AP b. PA radiograph of the right shoulder depicting degenerative changes to the humeral head consistent with Charcot arthropathy (white arrows).

**Figure 2:**
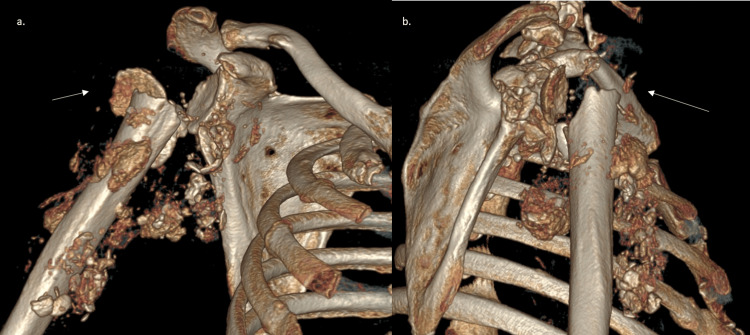
A 3D reconstruction scan showing destructive changes correlating with Charcot neuroarthropathy a. anterior view b. lateral view showing humeral head changes (white arrows).

**Figure 3:**
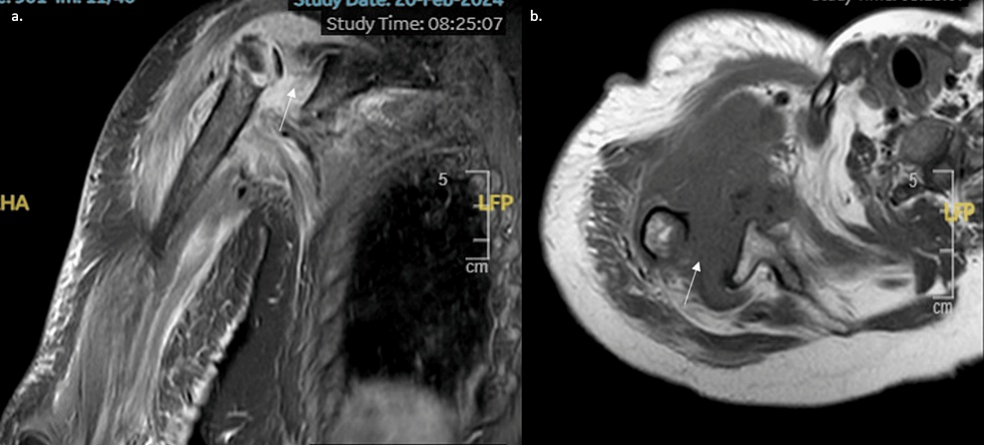
Magnetic Resonance Imaging radiographs prior to surgery a. Coronal b. Sagittal MRI view of the shoulder showing destruction of the shoulder joint (white arrows).

**Figure 4:**
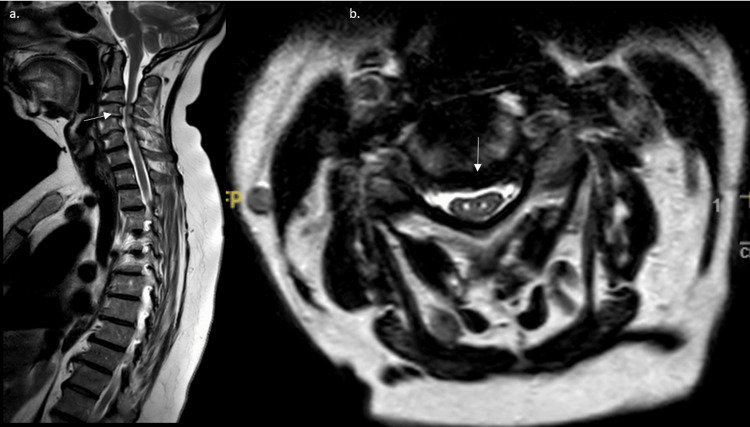
Magnetic resonance imaging of the spine a. T2 sagittal MRI of the spine showing cervical stenosis at the level of C3-4 (shown in white arrow). There is also evidence of lower-level impingement in the thoracic spine. b. Axial MRI of the spine with the “snake eye sign” shows extensive destruction and long-standing chronic changes in the spinal cord (white arrow).

Conventional radiography of the right shoulder showed a four-part fracture dislocation of the proximal humerus. This was partly due to a large joint effusion (Figure 1, 2). An MRI scan of the shoulder confirmed the comminuted fracture of the proximal humerus but also showed secondary destructive arthropathy of the shoulder joint (Figure 3). MRI scans of the cervical and lumbar spine showed severe cervical spondylosis with extensive myelopathy from C3 to C6 (Figure 4). Mild scoliosis of the lumbar spine was also seen with mild central canal and cervical foraminal stenosis although there was no critical narrowing of the exiting nerve root.

**Figure 5:**
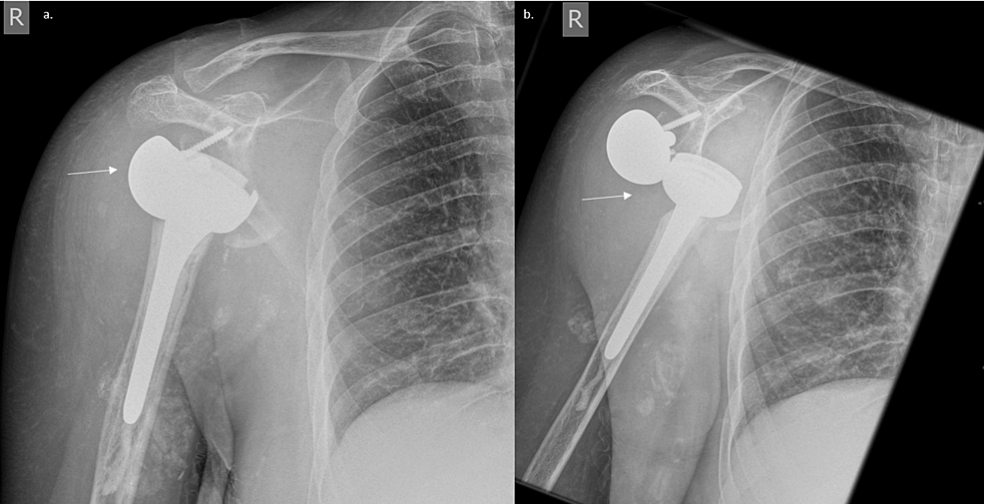
Radiograph of shoulder post-reverse total shoulder arthroplasties (TSA) a. AP of the prosthesis b. PA of the prosthesis The shoulder x-ray shows the dislocation of the reverse arthroplasty

A reverse total prosthetic replacement of the shoulder was performed. However, radiographic imaging in a review clinic at two weeks showed a dislocated prosthesis (Figure 5). An open reduction was performed where a glenoid fracture due to trauma was seen therefore the implant was removed and a washout was performed with microbiology sampling. The humeral stem was well fixed and left in situ as humeral osteotomy would have been required to remove this component. Currently, it has been a month since the surgeries. The current decision has been made to await healing of the glenoid fracture and a reassessment will be made if a reconstruction is feasible. 

## Discussion

Literature review

**Figure 6:**
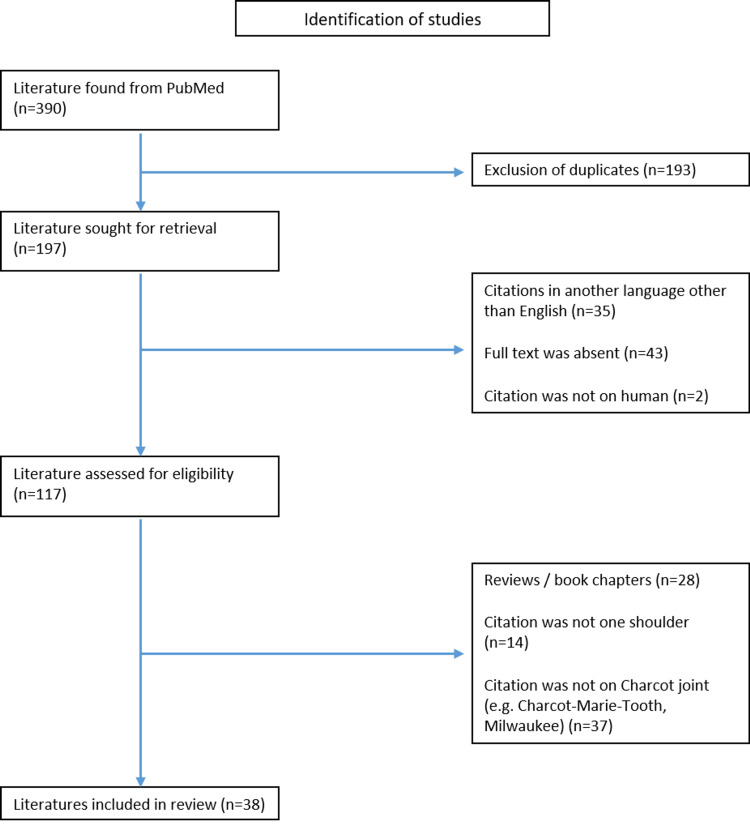
PRISMA flowchart showing the selection criteria

A literature review following the Preferred Reporting Items for Systematic Reviews and Meta-Analyses (PRISMA) guidelines was conducted, using the PubMed database by using Rayyan (Figure 6). The review was conducted on case reports with the terms “Charcot neuroarthropathy”, “Charcot arthropathy”, and “Charcot” combined with the term “shoulder” using the Boolean operator "AND”. A more detailed search with the terms “treatment” and “surgical” accompanying “Charcot arthropathy of the shoulder” with the Boolean operator “AND”.

Any literature that covered isolated arthropathy of the elbow, foot, and hand, along with Milwaukee shoulder syndrome and Charcot-Marie-Tooth were excluded. Any reports done in a language other than English were also excluded, along with duplicate publications and unavailable articles. A total of 159 articles were excluded and 38 articles with 58 cases were found to report cases of Charcot neuroarthropathy of the shoulder.

The characteristics of the cases are shown in Table 1. The mean age of the cases was 57.2 years (24-82 years), with an even distribution of male [n=27 (47%)] to female [n=31 (53%)] cases. There was no clear difference between the side of the shoulder the neuropathy presented with 25 (43%) right shoulders, 18 (31%) left shoulders, and five (9%) bilateral shoulders.

**Table 1:** Case reports of Charcot neuropathy of the shoulder RA: Rheumatoid arthritis, CSM: cervical spondylotic myelopathy, SM: syringomyelia, PFD: posterior fossa decompression, BP: brachial plexopathy, TSA: total shoulder arthroplasty, RSA: reverse shoulder arthroplasty. ROM: range of motion.

Article	Age	Sex	Side	Presentation	Cause	Treatment	Outcome
Luo et al. [6]	79	F	Bilateral	Pain Symmetrical supraspinatus + deltoid muscle atrophy Tenderness at acromion	RA	Conservative	N/A
Shi et al. [5]	82	M	Right	N/A	CSM	Unknown	N/A
Kocyigit et al. [7]	68	F	Right	Limited movement Numbness Burning Stiffness	SM	Conservative	Improvement of shoulder ROM (after 3 month follow up)
Wawrzyniak et al. [8]	76	F	Left	Swelling Weakness	SM	RSA	Relief of symptom Improved ROM
-	58	F	Right	Pain Swelling	SM	RSA	Relief of symptom Improved ROM
-	70	F	Right	Pain Swelling Weakness	SM	Conservative	Relief of symptom Improved ROM
-	69	F	Bilateral	Weakness Pain Swelling	SM	Conservative	Partial relief of symptoms
-	68	F	Right	Pain Swelling Weakness	SM	Conservative	Relief of symptom
-	72	F	Left	Pain Weakness	SM	Conservative	Partial relief of symptoms
-	68	F	Left	Pain	SM	Conservative	Partial relief of symptoms
-	64	F	Left	Pain Swelling Weakness	SM	Conservative	No relief
-	24	F	Left	Pain Weakness	SM	Conservative	No relief
-	61	F	Right	Pain Swelling Weakness	SM	Conservative	Partial relief of symptoms Improved ROM
Mahmoud et al. [9]	43	F	Right	Swelling Lmited movement	SM + Chiari type 1	N/A	N/A
Hirsch et al. [10]	47	M	Left	Discomfort Loss of sensitivity	SM + Chiari type 1	N/A	N/A
Choudhury et al. [11]	48	M	Right	Pain Swelling Decreased movement	SM	Conservative	Improvement in pain Mobility
Nambiar et al. [12]	29	M	Right	Pain Swelling	SM + Chiari type 1	PFD	N/A
Wang et al. [13]	52	F	Left	Pain Limited movement	SM + basilar impression	PFD	Relief of symptoms Reduced size of syrinx
Bocca et al. [14]	39	M	Left	Pain Swelling Limited movement	SM + Chiari type 1	N/A	N/A
Adiyeke et al. [15]	47	M	Left	Pain	SM	Conservative	N/A
Schoch et al. [16]	Mean age 64	M	N/A	N/A	BP	HA	Better
-	-	M	N/A	N/A	SM	HA	No change
-	-	M	N/A	N/A	SM	HA	Better
-	-	M	N/A	N/A	BP	HA	No change
-	-	M	N/A	N/A	SM	HA	Better
-	-	M	N/A	N/A	Peripheral neuropathy	HA	Better
-	-	M	N/A	N/A	Diabetes	TSA -> RSA	No change
-	-	M	N/A	N/A	Parkinsons	RSA	Better
-	-	F	N/A	N/A	Idiopathic	RSA	Better
-	-	F	N/A	N/A	Idiopathic	RSA -> HA	Worse
Nguyen et al. [17]	68	F	Right	Numbness Pain	Sensory neuropathy due to Primary Sjogrens syndrome	Conservative	No improvement
Su et al. [18]	71	M	Right	Numbness Tingling Swelling Pain	SM	Conservative	N/A
Kim [19]	52	F	Right	Pain Swelling	Tetraplegia	N/A	N/A
Chakraborty et al. [20]	40	F	Left	Discomfort Restriction of movement	SM	Conservative	N/A
Makihara et al. [21]	50	F	Left	Difficulty elevation Numbness	SM + Chiari type 1	Suboccipital decompression	Improvement of symptoms
Butala et al. [22]	53	F	Left	Stiffness Paraesthesia Weakness	SM	Conservative	Reduction of symptoms
Liu et al. [23]	44	M	Left	Swelling Limitation of motion	SM + Chiari type 1	Conservative	N/A
Alai et al. [24]	49	M	Right	Swelling Pain Weakenss Numbness	SM + Chiari type 1	Neurolysis + synovectomy	Pain relief Improved range of motion
Panagariya et al. [25]	62	M	Bilateral	Swelling Restriction of movement	SM	Conservative	N/A
Matsuhashi et al. [26]	54	F	Right	Swelling Pain Numbness	SM	Humeral head replacement	Improvement of symptoms
-	55	M	Right	Swelling Restriction of ROM	SM	Humeral head replacement	Pain remained Swelling reduced
-	64	F	Right	Pain Swelling	SM	Humeral head replacement	Decrease of pain Improved ROM
Gaskins et al. [27]	52	F	Right	Swelling Loss of mobility	SM + Chiari type 1	Suboccipital decompression Conservative	Pain improvement
Panda et al. [28]	56	M	Left	Pain Numbness Paraesthesia Loss of mobility	SM	Conservative	N/A
Grahovac et al. [29]	62	F	Left	Swelling Numbness Paraesthesia	SM + Chiari type 1	Suboccipital craniotomy	No progression
Murray [30]	73	F	Right	Weakness Swelling	SM	Conservative	N/A
Kumar et al. [31]	38	F	Right	Swelling Restriction of movement Paraesthesia	SM + Chiari type 1	Conservative	N/A
Nacir et al. [32]	54	M	Left	Limited ROM	SM + Chiari type 1	N/A	N/A
Garg et al. [33]	42	M	Right	Swelling Restricted ROM	SM + Chiari type 1	Arthrodesis	N/A
Crowther et al. [34]	40	F	Bilateral	Pain Sensory loss	SM	Shoulder resurfacing arthoplasty	Relief of symptoms
Kirksey et al.[35]	65	M	Right	Weakness Numbness	SM + Chiari type 1	N/A	N/A
Edison et al.[36]	65	F	Right	Decreased ROM Swelling	SM + Chiari type 1	N/A	N/A
Tristano et al. [37]	26	F	Right	Pain Swelling	SM	Arthrodesis	N/A
Cullen et al. [38]	36	M	Left	Pain Weakness	SM	N/A	N/A
Turkiewicz et al.[39]	80	M	Right	Pain	SM	N/A	N/A
Yanik et al. [40]	43	M	Left	Swelling Pain Loss ROM	SM + Chiari type 1	Arthrodesis	N/A
Louthrenoo et al. [41]	49	M	Bilateral	Swelling Numbness	SM	Conservative	Some improvement
Hwang et al. [42]	72	F	Right	Pain Numbness Weakness	SM + Chiari type 1	Conservative	Improvement

The majority of the cases that had been reported in the literature had reported that syringomyelia was the cause of neuropathy of the shoulder [n=47 (81%)].

Treatment methods varied with conservative treatment [n=24 (41%)] and surgical treatment [n=25 (43%)]. The treatment methods were not described in 10 cases. A variety of different methods were used in conservative treatment including medications, rehabilitation, physiotherapy, education, and immobilization. Amongst the 24 cases of conservative treatment, 11 cases (46%) showed improvement in symptoms, whereas three cases (13%) showed no signs of improvement. Surgical interventions included many different procedures mainly reverse shoulder arthroplasty (n=5) and hemiarthroplasty (n=6). Amongst the cases of surgical treatment, 15 cases (60%) relieved symptoms and range of motion, whereas six cases (24%) showed no improvement, with one case having worsening symptoms.

Charcot arthropathy is a rare chronic degenerative disorder that causes progressive destruction of joints, which was first described by French neurologist Jean-Martin Charcot who described inflammation and destruction of joints following denervation [43]. It can affect different joints ranging from the feet to the shoulders and the wrist. In Charcot arthropathy of the shoulder, according to a systematic review conducted by Rickert et al., patients in general present with reduced range of motion, loss of sensation, swelling, and weakness of the joint [44]. Patients may present with painful joints, whereas some present with painless joints, as confirmed by the systematic review [2, 44].

There are many different causes of Charcot arthropathy of the shoulder such as syphilis, diabetes, chronic alcoholism, and leprosy. However, through a review of previous literature, it can be said that the most common cause of arthropathy of the shoulder is syringomyelia [12, 27]. This was also confirmed by the systematic review conducted by Rickert et al., where the presence of a syrinx was detected in the majority of cases of Charcot's shoulder [44]. Very few literatures report Charcot arthropathy secondary to cervical spondylotic myelopathy. Cervical spondylotic myelopathy is a non-traumatic, progressive disease that causes degenerative changes to the vertebrae, intervertebral discs, and associated ligaments [45]. The majority of the previous literature reporting Charcot arthropathy secondary to cervical spondylotic myelopathy is associated with the hip and wrist joint [3, 4]. Only one report by Shi et al. describes the destruction of the shoulder joint accompanied by Charcot arthropathy of the feet [5].

The main treatment for Charcot arthropathy of the shoulder consists of conservative treatment. This includes the use of analgesia, nonsteroidal anti-inflammatory drugs (NSAIDs) to reduce inflammation, and physiotherapy to help range of motion [46]. Other techniques can be used conservatively to restrict weight bearing and immobilize the joint. However, a recent systematic review conducted by Wawrzyniak concluded that physical therapy could help improve the symptoms of pain in Charcot arthropathy of the shoulder, but it does not improve the range of motion of the shoulder [47].

In cases where the underlying cause of the Charcot joint is known, especially syringomyelia, treatment of the cause is known to improve symptoms. According to the literature, decompression of syrinx has been shown to slow joint deterioration and improve bone quality [2, 29, 48]. In a different case report by Wang et al., 15 out of 19 patients with Charcot's shoulder who had undergone surgery for the underlying syringomyelia showed neurological improvement with no further deterioration of the joint [13].

The research into surgical management of Charcot's shoulder has shown mixed results. Previously Matsuhashi et al. reported three patients with Charcot shoulder who had undergone total shoulder replacements in which all three patients experienced pain relief and significant improvement in range of motion [26]. On the other hand, a report by Wang et al. described how total replacement arthroplasty has been disappointing due to the high risk of recurrence and infection [13]. The systematic review conducted by Wawrzyniak et al. suggests that hemiarthroplasty has shown to have some success but in most cases has been shown to cause rotator cuff failures due to the inflamatous and destructive nature of the joint [47]. In cases where there has been severe bone destruction, instability, or damage to soft tissue including the rotator cuff muscles, many orthopedic surgeons have chosen to undergo reverse shoulder arthroplasty, which seems to be the current gold standard; provided there is sufficient bone stock, and a preserved deltoid function [47]. However, severe glenoid bone loss is a contraindication for the process. Ueblacker et al. report a case where they had performed bilateral reverse shoulder arthroplasties, with improvements to range of motion, stability, and function when followed up two years later [49]. However, this case supports the contrary argument that reverse shoulder arthroplasty is not a definitive solution for destructive arthropathy of the shoulder.

In this particular case, the patient was in pain with no obvious tumor or infection along with a history of low-velocity injury. The plan was to assess intra-operatively and debride if there was any infection, and a reverse total shoulder replacement was performed. However, due to the prosthesis being displaced two weeks post-operation, the implant was removed via open reduction, and a fracture of the glenoid was seen post-trauma. The fracture is currently being allowed to heal, in which the bone stock will be re-evaluated and revision surgical options are being explored which may include an augmented glenoid baseplate for a more robust fixation and to account for poor bone stock.

## Conclusions

This case report reports a rare case of Charcot arthropathy of the shoulder secondary to cervical spondylotic myelopathy following a low-velocity trauma injury. Although this cause of Charcot arthropathy is rare, it is important to consider in the atypical presentation of acute trauma. This report may provide valuable insight into the importance of imaging the cervical spine whilst evaluating new cases of Charcot arthropathy and the pitfalls of arthroplasty as definitive management.
